# Wearables: An R Package With Accompanying Shiny Application for Signal Analysis of a Wearable Device Targeted at Clinicians and Researchers

**DOI:** 10.3389/fnbeh.2022.856544

**Published:** 2022-06-23

**Authors:** Peter de Looff, Remko Duursma, Matthijs Noordzij, Sara Taylor, Natasha Jaques, Floortje Scheepers, Kees de Schepper, Saskia Koldijk

**Affiliations:** ^1^Behavioural Science Institute, Radboud University, Nijmegen, Netherlands; ^2^De Borg, Den Dolder, Netherlands; ^3^Fivoor Science and Treatment Innovation, Den Dolder, Netherlands; ^4^Shintō Labs, Eindhoven, Netherlands; ^5^Department of Psychology, Health and Technology, University of Twente, Enschede, Netherlands; ^6^Affective Computing Group, Media Lab, Massachusetts Institute of Technology, Cambridge, MA, United States; ^7^PsyData Group, Department of Psychiatry, UMC Utrecht, Utrecht, Netherlands

**Keywords:** wearables, heart rate, electrodermal activity, R Shiny application, neuroscience, treatment, physiological reactivity

## Abstract

Physiological signals (e.g., heart rate, skin conductance) that were traditionally studied in neuroscientific laboratory research are currently being used in numerous real-life studies using wearable technology. Physiological signals obtained with wearables seem to offer great potential for continuous monitoring and providing biofeedback in clinical practice and healthcare research. The physiological data obtained from these signals has utility for both clinicians and researchers. Clinicians are typically interested in the day-to-day and moment-to-moment physiological reactivity of patients to real-life stressors, events, and situations or interested in the physiological reactivity to stimuli in therapy. Researchers typically apply signal analysis methods to the data by pre-processing the physiological signals, detecting artifacts, and extracting features, which can be a challenge considering the amount of data that needs to be processed. This paper describes the creation of a “Wearables” R package and a Shiny “E4 dashboard” application for an often-studied wearable, the Empatica E4. The package and Shiny application can be used to visualize the relationship between physiological signals and real-life stressors or stimuli, but can also be used to pre-process physiological data, detect artifacts, and extract relevant features for further analysis. In addition, the application has a batch process option to analyze large amounts of physiological data into ready-to-use data files. The software accommodates users with a downloadable report that provides opportunities for a careful investigation of physiological reactions in daily life. The application is freely available, thought to be easy to use, and thought to be easily extendible to other wearable devices. Future research should focus on the usability of the application and the validation of the algorithms.

## Introduction

Physiological signals, such as heart rate (HR) and skin conductance [i.e., electrodermal activity (EDA)], are increasingly being used in clinical practice and healthcare research (Regalia et al., 2019; Johnson and Picard, 2020). They provide innovative ways to bring neuroscience from the lab to real-life settings (Johnson and Picard, 2020). Researchers study associations between physiological signals and a plethora of emotional, cognitive, and physical diseases, such as epilepsy (Regalia et al., 2019), depression (Pedrelli et al., 2020), burnout (de Looff P. C. et al., 2019), suicide, aggressive behavior (de Looff P. et al., 2019; Goodwin et al., 2019; de Looff et al., 2022), or mood (Sano et al., 2018; Umematsu et al., 2019). These wearable devices can also be used by clinicians and researchers to study psychological-physiological associations in day-to-day and moment-to-moment situations, which is known as digital phenotyping (Onnela and Rauch, 2016).

Traditionally, the physiological signals were validly measured in the lab, but wearable technology provides increased utility for real-life (Johnson and Picard, 2020), and that is where it matters most. Wearable devices have the potential to bring the lab to the daily life of many users and are increasingly creating an impact on the management of many problems and diseases (Johnson and Picard, 2020). Physiological data from wearables can for instance be used to monitor disease (Regalia et al., 2019), predict risk for disease (Pedrelli et al., 2020), or provide continuous feedback on bodily signals to stimulate emotional awareness and, subsequently, behavioral change (Derks et al., 2019).

**FIGURE 1:**
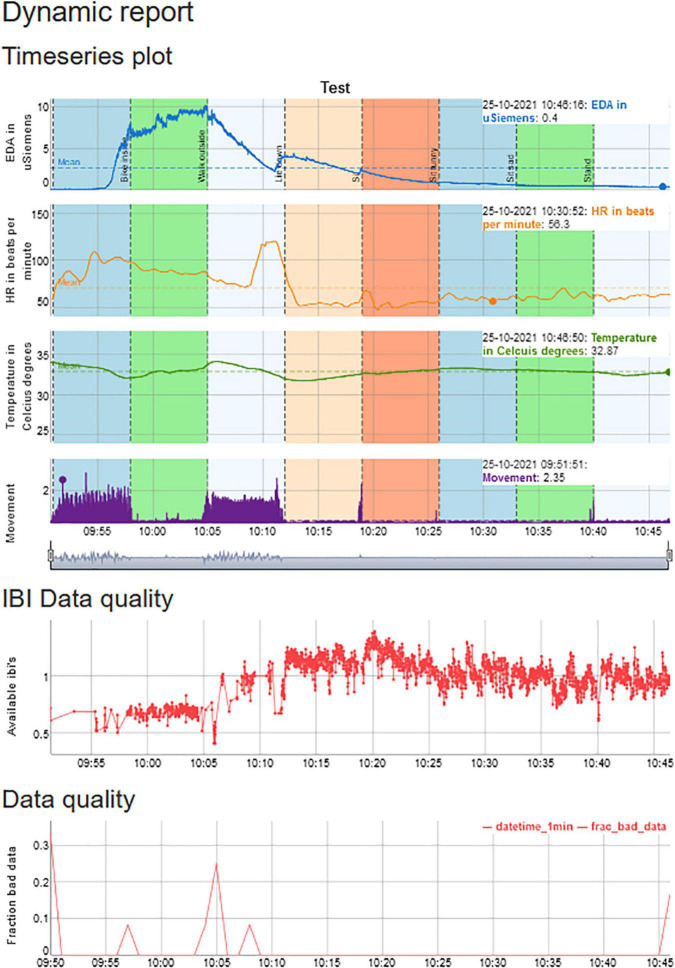
An example of a session that contains physiological data on EDA, HR, temperature, and movement, but also provides information from HR, movement, and EDA on the number of artifacts.

Clear guidelines exist on processing signals such as HR and EDA and are performed with the utmost rigor in laboratory settings (Task Force Electrophysiology, 1996; Society for Psychophysiological Research *Ad Hoc* Committee on Electrodermal Measures, 2012). Signal processing in real-life situations is somewhat more challenging (see Figure 1), especially for wrist-worn devices (Cosoli et al., 2020).

**FIGURE 2:**
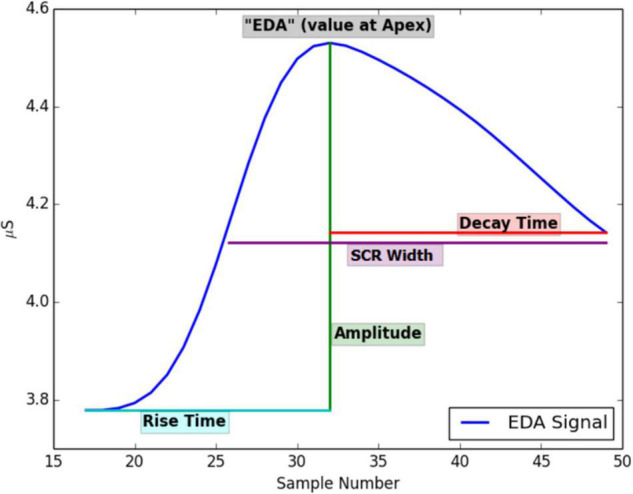
Features that can be extracted from an EDA signal. Printed with permission from Taylor et al. (2015).

Although data collection with the wearables is typically straightforward, signal processing (i.e., pre-processing, artifact detection, and feature extraction) of the physiological data is often without formal guidelines (Coffman et al., 2020). The absence of guidelines makes it challenging to use wearables, especially for researchers and clinicians who did not receive formal training in signal processing and analysis. An additional challenge is that signals obtained with wearables, such as HR, EDA, temperature, and movement, can suffer from large amounts of artifacts (i.e., noise) due to electrode placement, loose electrodes, sensor failure, movement, or skin tone, among other factors (van Lier et al., 2019; Schuurmans et al., 2020). Most physiological signals should, therefore, preferably be cleaned, filtered, and modeled using various techniques depending on the physiological signal. For instance, EDA is often pre-processed by up- or downsampling, signal smoothing, and artifact detection using various (rule-based) algorithms (Bach, 2014; Taylor et al., 2015; Coffman et al., 2020). Subsequently, various features are extracted from the EDA signal that can, for instance, include the tonic skin conductance level, or phasic changes in skin conductance (called peaks). Phasic changes can be determined by calculating the amplitude, width, rise time, or decay time of the peak, as shown in Figure 2 (but for an excellent overview of EDA features see Boucsein, 2012).

The wearables data also provides clinicians with several opportunities in treatment settings (Johnson and Picard, 2020). Clinicians might want to use the information obtained with the wearables to discuss and understand the physiological reactions to real-life stressors, events, and situations with their patients or explore the physiological reactions to stimuli in therapy sessions (Derks et al., 2019; Looff et al., 2021). Clinicians might also want to conduct their research to investigate if therapy affects physiological reactivity. However, mapping and synchronizing physiology and psychological events and situations are troublesome and clinicians often have no formal training in signal processing, which makes it difficult to assess the validity and reliability of physiological signals and underlying algorithms for artifact detection and feature extraction (Menghini et al., 2019; van Lier et al., 2019; Milstein and Gordon, 2020; Schuurmans et al., 2020).

In the current paper, we describe the creation of a Shiny application (Shiny is a tool for data visualization) with an accompanying R package (called “wearables”) that might prove useful for clinicians and researchers who work with the Empatica E4, although the Shiny application and R-package can easily be extended to signal analysis with other devices. The Empatica E4 was chosen as the data is easily accessible and provides raw data on most physiological signals (Poh et al., 2010). R was chosen as it can be used to create beautiful visuals in an open-source framework, which ensures that it is available to a large population of users (R Core Team, 2014; R Studio, 2021).

In contrast to the Empatica E4, commercial devices from technology companies often provide dashboards with a multitude of data insights for reasonable prices, while data is only provided in the interpreted form, without openness on the underlying algorithms or definitions (e.g., “body battery,” “stress coping score,” and “steps”). Also, concerns arise about privacy, and judicial implications as data are transferred non-anonymized to company servers, which is especially troublesome when working in healthcare (van den Braak et al., 2021). We, therefore, set out to analyze the data locally on a computer and create an interface that would allow researchers to batch process large amounts of data. It also provides clinicians with tools to visualize the physiological data and synchronize the physiological data with real-life events using a calendar. Clinicians would preferably also have the opportunity to extract useful features over periods that they want to analyze.

The scripts that are used in the current study were originally utilized for studies into the relationship between physiological predictors of aggressive behavior of forensic patients with mild to borderline intellectual functioning (de Looff P. et al., 2019) and the relationship between physiological predictors of burnout in health professionals who work with these patients daily (de Looff P. C. et al., 2019). The data from those studies consisted of ∼5 days of physiological data from ∼100 patients and ∼100 professionals. Signal analysis in the form of pre-processing, artifact detection, and feature extraction were carried out both visually and automatically with a combination of R (R Core Team, 2014) and Python code (Rossum and Team, 2018), as the algorithms were written in both R and Python and available in various packages. The bridge between R and Python code was somewhat troublesome and considerable knowledge of programming and coding was required to handle the data. To increase the unity and ease of use for non-technical users, but also to stimulate open-source science and reuse of the code for these complex physiological data, we set out to create a Shiny application with an R package that can be used by both clinicians and researchers. During the studies on aggressive behavior in patients and burnout in professionals, it became apparent that not only researchers wanted to work with the data from the wearables, but health professionals as well. Health professionals recognized several opportunities for using the data in the daily life of patients and during treatment to study physiological reactions over the day, during situations and events, or in therapy. Therefore, we set out to create an application that can be used to visualize the signals, synchronize calendar data with physiological data using annotations, detect noise and artifacts, extract features, and automatically batch process large amounts of data. In the studies that were conducted over the past years (de Looff P. et al., 2019; Looff et al., 2021) and a study that is currently in preparation, the participating staff members and patients provided feedback on the use of the Empatica E4, the accompanying software, provided comparisons with other hard- and software and gave us feedback on earlier versions of the E4 dashboard following a user-centered design approach. Note however that no formal study on the usability of the current version of the application has been conducted.

In sum, we set out to create a Shiny “E4 dashboard” application with an accompanying “Wearables” R package to visualize and report on the physiological data including a calendar. The software is targeted at clinicians who work with physiological data and have limited knowledge of signal analysis. In addition, for researchers, a batch processing application is available to pre-process, detect artifacts, and extract features. To our knowledge, this is the first open-source R package with a Shiny application that simultaneously processes the physiological signals, offers flexible pre-processing, artifact detection, and feature extraction, and has several visualization tools for expert and non-expert users.

The current paper first discusses the Empatica E4 device, the physiological signals that can be obtained with the E4, and the implementation in the method section. The results section provides an overview of the functionality of the Shiny application and wearables package. Lastly, in the discussion we provide an overview of the advantages, limitations, directions for future research, and clinical implications of working with the Empatica E4, Shiny “E4 dashboard,” and “Wearables” R package.

## Method

### Empatica E4

The wearable Empatica E4 can be used to record physiological signals over the day and offers two modes of recording: (1) real-time *via* an app, or (2) the user can store the data locally on the device. In (1) real-time, Empatica offers an application in which the physiological data is displayed on Android or IOS devices. After finishing the real-time recording, the data is transferred to Empatica Connect (i.e., the cloud solution from Empatica) *via* a mobile or Wi-Fi internet connection. The recording can also be stored on the (2) local memory of the Empatica E4. When the recording is finished, the data is transferred *via* USB with an application called E4 manager that downloads the data from the Empatica E4 and uploads it to Empatica Connect. The Empatica E4 data can be visualized, deleted, or downloaded on Empatica Connect.

Empatica offers physiological signals in raw data format (e.g., EDA, blood volume pulse, temperature, and movement) or processed format (e.g., HR and interbeat interval), and offers a visualization feature. However, Empatica offers no tools for signal analysis or visualization mapping of psychological-physiological associations other than timestamp data. Timestamps can be made with a button that is on the E4 case and is stored as the time on which the button is pressed. On Empatica Connect, the timestamps are visualized as red lines in the graphs. The physiological data can then be downloaded as a zip file and contains the physiological signals in .csv files. The zip files can be stored on your local computer or network for further analysis.

### Electrodermal Activity

The EDA from the Empatica E4 is measured with dry electrodes that detect changes in the electrical conductivity of the skin (Poh et al., 2010; Garbarino et al., 2014). The EDA signal is known to be influenced by skin temperature and motion (Coffman et al., 2020) and should be taken into account when modeling the data, which is possible as the Empatica E4 records these as well. EDA is sampled at a frequency of 4 Hz and is measured in uSiemens (Garbarino et al., 2014; Milstein and Gordon, 2020). The EDA signal is stored in a CSV file (EDA.csv) that contains the start of the recording in the first row in Unix time (i.e., in Universal Time Coordinated (UTC). Unix time is the number of seconds elapsed since January 1, 1970). The second row contains the sampling frequency (e.g., 4 Hz for the EDA signal). Subsequently, the EDA signal (from row three onward) is stored in the remainder of the column, in which each row represents 250 ms of data (i.e., four rows, thus, represent one second of data). The resolution of one digit is approximately 900 pSiemens and the signal ranges from 0.01 uSiemens up to 100 uSiemens. EDA is thought to primarily be the result of sympathetic innervation (Boucsein, 2012) and the EDA signal is preferably pre-processed, checked for artifacts that can be discarded for analysis, and features, containing characteristics of the signal, can be calculated (Boucsein, 2012), following the guidelines of skin conductance processing (Society for Psychophysiological Research *Ad Hoc* Committee on Electrodermal Measures, 2012).

The signal is typically decomposed into tonic and phasic components (Coffman et al., 2020). Tonic components change slowly while phasic components change more rapidly as a result of demands, such as psychosocial or biogenic stressors (Boucsein, 2012; Everly and Lating, 2019). Features (see Figure 2) are, for instance, the tonic skin conductance level (SCL), or phasic changes in skin conductance that have the appearance of “peaks” (SCR) in the signal. From the EDA signal, peaks are detected, and several peak features are calculated: EDA at the start of the peak, rise time, maximum derivative, amplitude of the peak, decay time, SCR width, and area under the curve (AUC) (for an overview of the features and abbreviations see Boucsein, 2012, p.2). The detected peaks (non-specific skin conductance responses) are further used to calculate peaks per minute. We utilize a script to analyze the skin conductance signal that was created by two of the co-authors of the current study (Taylor et al., 2015), which was written in version 2.7 of the Python language (Rossum and Team, 2018). In short, the EDA signal is first upsampled to 8 Hz; then, a Finite Impulse Response low pass filter is applied. A support vector machine algorithm that was trained using expert data is used to classify the data into artifact and artifact-free periods. Values approaching 0 are discarded in the software, following quality assessment procedures defined by Kleckner et al. (2018). Finally, peak detection is applied to the signal, and several peak features are calculated (see Taylor et al., 2015 for an overview) with a threshold of 0.005 uSiemens following the study by de Looff P. et al. (2019).

### Heart Rate

The HR is recorded with a photoplethysmography (PPG) sensor that emits green (i.e., light absorption is higher in oxygenated blood) and red light (i.e., to detect motion artifacts) and samples at a frequency of 64 Hz, with which a blood volume pulse (BVP) signal is obtained (Corino et al., 2017; Hayano et al., 2020). The resolution of the sensor output is 0.9 nW per digit. PPG sensors are used to measure volume changes in the blood. The PPG sensor provides a signal that can be used to calculate HR but can also be used to calculate other vital signs such as breathing rate and blood pressure (Orphanidou, 2018). Empatica utilizes two algorithms on the BVP signal to construct an inter-beat-interval, with which HR (and HR variability) can be calculated. The two algorithms are optimized to detect heartbeats and to discard beats that contain artifacts, but only the first algorithm is disclosed by Empatica (Schuurmans et al., 2020; Empatica support, 2021). The BVP signal (available from BVP.csv), thus, results in two additional CSV files that contain the inter-beat-interval (IBI.csv) and the HR data (HR.csv). Both the HR files and IBI files are used in the current application. Several HR variability (HRV) parameters can be calculated from the IBI, such as time-domain measures, frequency domain measures, and non-linear analysis (Kamath et al., 2016). Note that, although we refer to HR and HRV, studies also use pulse rate (variability) to refer to data that is obtained with a PPG sensor as opposed to heart rate variability to refer to data that is obtained with an electrocardiogram (Schäfer and Vagedes, 2013).

The BVP file consists of one column with Unix time in the first row, and frequency in the second row (64 Hz), followed by the BVP data. The IBI file consists of two columns with the Unix time in row 1. The first column contains the number of seconds that have elapsed since the start of the file, and of which the algorithm is certain that it detected a beat. The second column is the duration in seconds between consecutive beats (the IBI). The HR file contains one column with the Unix time in the first row, the frequency (1 Hz) in the second row, followed by the average HR per second, based on the BVP signal.

Several methods exist to detect artifacts that defy natural cardiac functioning. For instance, average HR typically does not exceed values between 40 and 180 beats per minute (although higher values might be feasible with exercise), which corresponds to a maximum R-R interval of 1.5 s (with 40 beats per minute). A common HR range would thus be 0.67 Hz (40 beats per minute) to 3 Hz (180 beats per minute) with 5 Hz (300 beats per minute) during exercise (Orphanidou, 2018). Lastly, the ratio of maximum R-R interval to the minimum interval is expected not to exceed 1.1 over a 10-s segment, as HR typically does not change by more than 10% under normal circumstances. Allowing a single missed beat would mean that the R-R interval can have a maximum value of 3 s and the ratio of maximum to minimum R-R interval would be 2.2 (Orphanidou, 2018). For incorporation in the Shiny application, the batch tool, and the R package we used an existing R package [RHRV; (Rodríguez-Liñares et al., 2011; Martínez et al., 2017)] to analyze HRV based on the IBI. RHRV removes ectopic beats, uses adaptive thresholding to remove questionable beats, and discards unlikely physiological values (see Martínez et al., 2017, p.21–22). HRV analysis is traditionally performed on the R-R interval (Orphanidou, 2018). However, measurements of BVP on the wrist are artifact-prone and often result in IBI files that present as “gaps” in the RR signal, as the Empatica algorithm discards beat that contain artifacts. Several methods exist to assess the quality of the signal and apply correction methods to beats (Orphanidou, 2018). The E4 dashboard implements several algorithms based on EDA, HR, and movement that provide the user with information on the amount of noise in the recording, but currently does not provide beat correction methods. This is in part due to the unavailability of the first part of the beat detection algorithm, and the absence of a wide array of pulse peak detectors (see Orphanidou, 2018). Thus, we would strongly urge users to only use HRV analysis on resting-state and non-movement conditions (Schuurmans et al., 2020).

### Movement

Movement with the Empatica E4 is sampled at a frequency of 32 Hz (and has a resolution of 8 bits) with a sensor that measures acceleration in space over time on an *x*, *y*, and *z*-axis (Milstein and Gordon, 2020; Schuurmans et al., 2020). The first row of the CSV file (ACC.csv) contains the starting time of the recording, the second row contains the frequency (32 Hz), and the subsequent rows consist of three columns that contain the raw acceleration on the x, y, and z-axis. The accelerometer measures acceleration in the range between −2g and 2g. For the current study, we calculate the mean magnitude of acceleration over the three axes (de Looff P. et al., 2019), which is given by:


$$\frac{\sqrt{x_{i}^{2}+y_{i}^{2}+z_{i}^{2}}}{64}$$

Note that division by 64 results in the mean magnitude of acceleration expressed in gravity force (Rowlands et al., 2015).

### Temperature

The Empatica E4 also contains an optical infrared skin temperature sensor that samples at a frequency of 32 Hz and a resolution of 0.02°C. The CSV file (TEMP.csv) contains the Unix starting time in the first row of the file, followed by the frequency of the recording in the second row. Subsequently the raw temperature data is available in Celsius degrees (Garbarino et al., 2014). If participants used the event marker of the Empatica E4, then, a tags’ file is also available with the Unix times of the events that were marked.

### Implementation

For the above described studies (de Looff P. et al., 2019; Schuurmans et al., 2020), we used the Empatica E4 (Poh et al., 2010; Garbarino et al., 2014; Picard et al., 2015; Taylor et al., 2015). To determine possible designs of the interface for clinicians, a mixed methods study was carried out to target features that were considered important for clinicians to include in an interface and that would increase the usability and acceptance of wearable biosensors in forensic psychiatry (Looff et al., 2021, and an article in preparation).

## Results

### Overview of Dashboard

#### Overview of Functions

The Empatica E4 dashboard contains six tabs for data analysis of a selected Empatica file (Data Tab, Calendar Tab, Visualization Tab, and Analysis Tab), one Tab for cutting files (Data Cutter Tab), and one Tab for batch processing multiple files (Batch analysis Tab).

**FIGURE 3:**
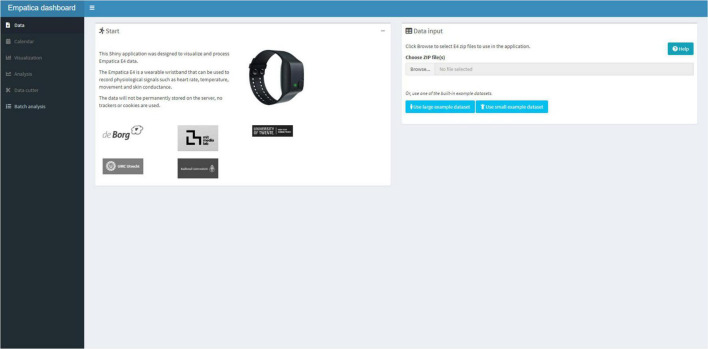
The data tab user interface, in which data can be read in from Empatica zip files.

To start, the Data Tab (Figure 3) is used to browse local folders to select the downloaded Empatica zip file that the user wishes to analyze. The data is uploaded to the local R session and read in as a list with prepended time columns. The user has the option to use two datasets with example data consisting of a large dataset that contains approximately 8 h of data and a smaller dataset with approximately 1 h of data. Note that the user also has the option to select multiple zip files recorded on the same day. This option was added as participants sometimes accidently turn off the device, which results in multiple files per day.

**FIGURE 4:**
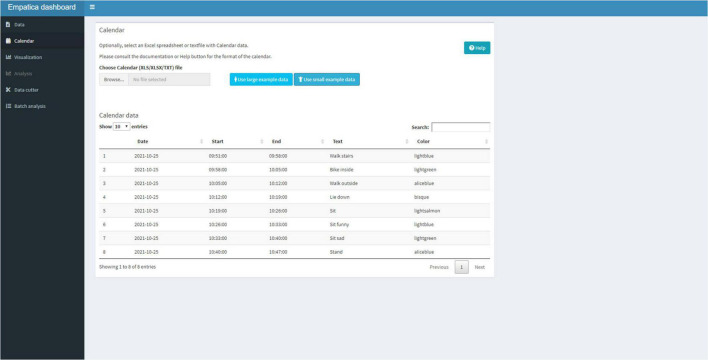
The calendar tab that can be used to add text to the visualization and color shade activities.

After successfully reading the E4 data, the user can move to the Calendar Tab (Figure 4) and upload a Text or Excel file that contains calendar information. The five columns need to be formatted into: a (1) Date (a day-month-year variable, note that R Shiny displays the date variable in year-month-day format), a (2) Start (an hour:minute:seconds variable), (3) an End (an hour:minute:seconds variable), the (4) Text (the text that needs to be displayed in the graph), and a (5) Color. The color names to be used for shading the graph are available under the “Help” button. The calendar is uploaded in the R session and can be viewed as a data table in the Shiny application.

**FIGURE 5:**
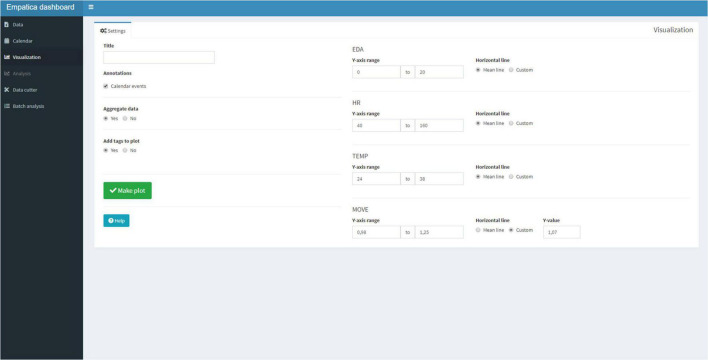
The visualization tab has a Settings Tab that can be used to choose the settings to be displayed. Preset ranges for the physiological signal are: EDA (0–20 micro Siemens), HR (40–160 beats), temperature (24–38°C), and movement (0.98–1.25 mean acceleration).

Subsequently, the Visualization Tab (Figure 5) can be selected. The Visualization Tab has a separate Settings Tab and a Plot Tab. In the Settings tab, the user can add a title to the plot, and checkmark if the annotations should be displayed. The user has the option to plot the raw data if the entire session lasted less than 2 h. If the session exceeds 2 h of data, then a 1-min aggregation is used to display the plot, for speed of use. Recall that the user was also able to create tags with the Empatica E4 to create event marks. If tags were created, the user has the option to display the tags. Lastly, the user has the option to change the default ranges of the graphs and can add a line to mark the mean or a custom value. Physiological parameters differ per person, per season, and even per day (Boucsein, 2012; Kamath et al., 2016; Orphanidou, 2018), and this offers the possibility to visualize the personal physiological parameters. If the app is used for a longer period, the user will get a good impression of what the typical resting EDA or HR is. A default means acceleration for movement is set at 1.07 to display periods with over 7% of mean acceleration, as movement can be a source of artifacts (Schuurmans et al., 2020).

**FIGURE 6:**
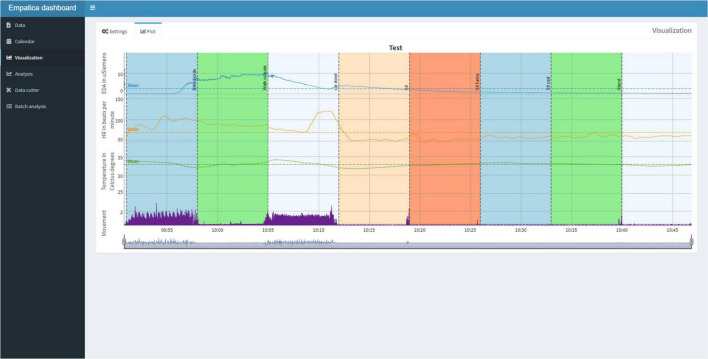
The Plot Tab can be used to visualize the physiological signals and synchronize them with the calendar events. A session is displayed for approximately 1 h, in which one of the authors undertakes eight activities, each lasting 7 min, to indicate the differences in physiological reactivity during various activities and illustrates differences in physiological reactivity.

Subsequently, the user can view the Plot tab (Figure 6) for the plots of the physiological signals in combination with the calendar events. If desired, the user can switch back to the Settings Tab to adjust the settings and replot.

**FIGURE 7:**
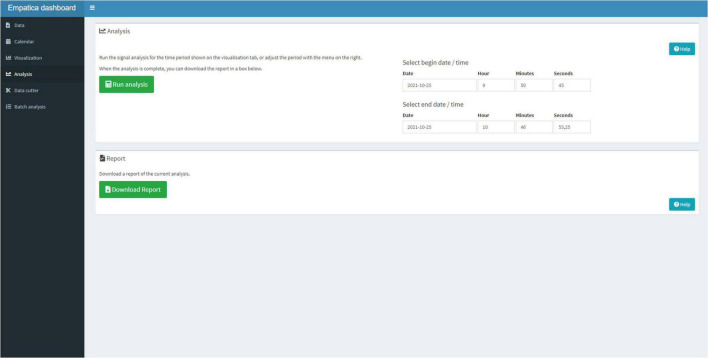
The Analysis Tab can be used to select a timeframe, for which the signal analysis should be performed and download a report.

Next, the user can select the Analysis Tab (Figure 7) to run the signal analysis scripts on the entire period or the user can select smaller periods that need to be analyzed. When the script finished its analysis, the user can download a Hyper Text Markup Language (HTML) report that contains the visualization, the calendar, and the analysis in a standardized format.

**FIGURE 8:**
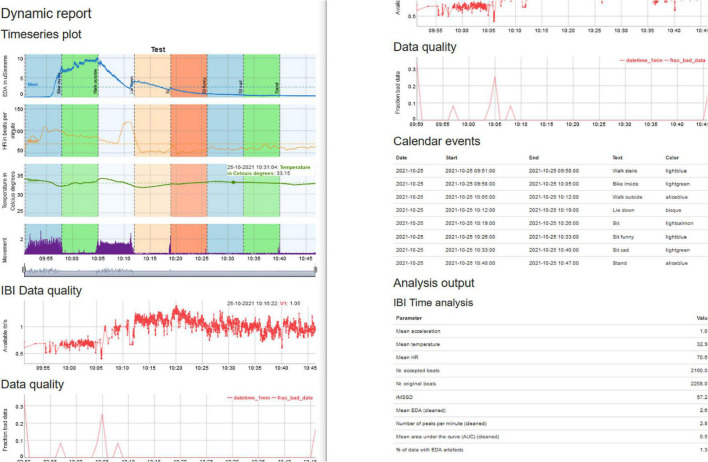
Summary report of the analysis that can be read with your local browser.

The HTML report in Figure 8 contains the graph, which can be interactively panned. It also includes information on the fraction of artifacts that are present in the data from both the IBI algorithm and the EDA artifact detection and provides the calendar that was uploaded. We have made a summary Table that contains the main parameters for EDA, HR, temperature, movement, and the percentage of artifacts that was present in the data.

#### Data Cutter Tab

To analyze the physiological data, researchers often use statistical techniques, such as multilevel modeling or machine learning, for which they need 1-min or 5-min interval bins (or any other interval) that can be used for analyses (de Looff P. et al., 2019; Goodwin et al., 2019; Pedrelli et al., 2020) to, for instance, study epilepsy (Regalia et al., 2019), depression (Pedrelli et al., 2020) or emotion regulation (Derks et al., 2019). The data cutter can be used to select a timeframe that needs to be cut and the interval bins, in which this is needed. For instance, recall that the study conducted by de Looff P. et al. (2019) used multilevel analyses over a 30-min timeframe in 5-min interval bins preceding aggressive incidents. The physiological data were, thus, divided into 6-time frames with 5-min interval bins to study the physiological trend preceding aggressive behavior. The output of the data cutter is a ZIP file that is similar to the Empatica E4 ZIP files, and can be loaded into the Shiny application or used in the batch analysis.

#### Batch Processing Option

If a clinician or researcher wants to batch process larger amounts of data, the batch processing option is available. The user can select an input folder where all ZIP files are stored and can select an output folder, where all the processed ZIP files must be saved. The processed data inherits the name of the ZIP file to ensure that the processed data can be traced back to the physiological data. This results in an RDS file (a native R file) that contains the parameters that were calculated. The summarized parameters in the HTML file are quite limited and intended for non-experts. The parameters in the RDS file are quite extensive and intended for more experienced users. The RDS file contains the raw E4 data, the signal processing data frames, extracted features of the physiological signals, and often used metrics, such as the mean, min, max, and median of the physiological data over the specified timeframe.

#### Signal Analysis (Pre-processing, Artifact Detection, Feature Extraction)

##### Pre-processing

Both single file analysis and batch file analysis follow a similar protocol for processing physiological signals. A ZIP file is temporarily unzipped and read into a list containing the raw physiological data. First, timestamp columns (in human-readable time) are prepended to the physiological datasets. Starting time is determined based on the Unix time of the E4-files, and is timestamped based on the system time of the user. This enables the users to write down events in their time zone and synchronize the calendar data with the physiological events. The EDA signal is upsampled to 8 Hz, and one column is added to the ACC (movement) data that contains the mean acceleration. The IBI file is somewhat different from the other files in that it contains the first column with the Unix start-time in the first row and the number of seconds elapsed since the start time that a heartbeat was detected. The second column contains the duration of the inter-beat-interval.

##### Artifact Detection

Following pre-processing, the EDA signal is checked for artifacts following the algorithm developed by Taylor et al. (2015). In short, several features are extracted from the EDA signal, which is put into an algorithm that classifies for each segment of 5 s, whether this segment contains an artifact. The algorithm is a support vector machine pre-trained on expert data and implemented by Taylor et al. (2015). Calculations are performed on the segment and one-second and half-a-second wavelet decompositions of the measurements. The pre-training also determined which of the features are used in the support vector machine algorithm. The algorithm has two settings, binary classification (artifact and no artifact) and ternary classification (artifact, unclear, and no artifact).

In addition, besides artifact detection based on EDA, we also provide information on artifacts for HR. We used an existing R package for incorporation in the Shiny application and the R package (RHRV; (Rodríguez-Liñares et al., 2011; Martínez et al., 2017) to analyze HRV based on the IBI (but note that Empatica also applies an undisclosed algorithm on their BVP signal to detect heartbeats). The HR variability calculations are based on the IBI signal that contains the number of milliseconds since the start of the recording (Martínez et al., 2017). The IBI data is first used to build a non-interpolated heart rate series. Then, RHRV removes RR intervals that were too short or where missed beats were detected, which results in the number of accepted beats versus the number of original beats that were detected. The heartrate series are then interpolated to be used for time series analysis and frequency analysis. Note that to obtain HR variability parameters, the latest research indicated that these measures can only be interpreted under resting conditions (Menghini et al., 2019; van Lier et al., 2019; Schuurmans et al., 2020). The artifact detection algorithms from EDA, HR, but also movement, thus, provide the user with several indications of the trustworthiness of the physiological data.

##### Feature Extraction

Both the single file analysis and batch file analysis provide aggregations of the physiological signals. The single-file analysis is targeted at users that have no background in programming and are typically clinicians or researchers. The HTML output provides them with often used parameters of the physiological signals, such as the mean EDA, peaks per minute, HR, RMSSD, temperature, and movement. The batch file analysis is provided for more experienced users, such as researchers with some knowledge of programming and physiology. The native RDS files can be used for further analysis by the user and provide information on range values of EDA, HR, HRV, temperature, and movement, as well as the artifact detection and feature extraction data frames. Lastly, experienced users can also adjust the functions of the wearables package and the E4 dashboard to suit their needs or incorporate additional algorithms for pre-processing, artifact detection, or feature extraction. The Shiny application^1^ is available on Github, while the Wearables R package is available on CRAN^2^ and the latest version is also available on Github.^3^

##### Software Development

We have created several functions in the Wearables package that are used in the E4 dashboard. The functions are designed to handle specific objects from the Empatica E4, such as the CSV files that contain the physiological data, the sampling frequencies, and datetimes that accompany these files. The Wearables package also provides functions to further process the data frames of the physiological signals and apply several artifact detection and feature extraction algorithms to the objects. The E4 dashboard has a modular set-up, in which each Tab in the application has a separate module. For the development of the Wearables package and E4 dashboard, we have incorporated frequently used and stable packages and frameworks to ensure that there is a low risk of incompatibility with future versions of R or Shiny (Wickham, 2019).

## Discussion

With the current study, we have created an R package called “Wearables” and an accompanying R Shiny application called “E4 dashboard.” The software can be used by clinicians and researchers to visualize and (batch) analyze physiological signals that are obtained with the Empatica E4. To our knowledge, this is the first R package with an accompanying Shiny application that simultaneously processes physiological signal data, offers flexible signal pre-processing, artifact detection, and feature extraction (Orphanidou, 2018), and has a built-in visualization tool.

The main advantage of using the Shiny application to inspect collected and annotated data is the ability to construct a very detailed and personalized profile of the physiological responses to stressors, events, and situations that are considered to be of importance for a specific patient. This functionality allows clinicians to map the physiological reactivity of a patient over time, in specific day-to-day or moment-to-moment situations, or to investigate if a treatment affects physiological reactivity. The physiological values can be easily calculated as the signal analysis options are built into the Shiny application and uses preset parameters from frequently used packages (Taylor et al., 2015; Martínez et al., 2017). The clinician can also inspect graphs to determine whether there is increased movement or an increased number of artifacts in the data. This provides valuable information about the validity and reliability of the measurements (Menghini et al., 2019; van Lier et al., 2019; Milstein and Gordon, 2020; Schuurmans et al., 2020). The downloadable reports that are generated can serve as a talking board and as a basis to discuss physiological reactivity (both hypo- and hyperreactivity) in real-life and treatment settings. Clinicians (together with their patients) can investigate if changes over time and/or in specific situations are evident, serve as a method to quantify the physiological reactivity, and consider the reliability of the measures. The software thus provides opportunities for an in-depth discussion and careful investigation of physiological responses in daily life (Johnson and Picard, 2020).

The Shiny application provides users with a flexible method to visualize single files or batch process multiple files. This effectively means that the wearable can be used by clinicians and health researchers with little knowledge of signal processing. In addition, the software also allows for sufficient flexibility in the Wearables package for advanced analysis, as the code can simply be modified and adjusted by researchers, who need additional signal processing functionality in a popular and easy-to-use R Shiny framework (R Studio, 2021).

Another advantage of the current Wearables package and Shiny application is that the data of a popular wearable device can be processed locally without dependence on cloud-based solutions, which has important privacy advantages to handle physiological data (van den Braak et al., 2021), but can also easily be integrated into a local server or cloud-based solution if organizations would like to scale up to processing large amounts of data (Price and Cohen, 2019). It should however be noted that the anonymized physiological data from the Empatica is uploaded to the cloud and can only then be downloaded to a local machine.

### Limitations

The current Shiny application was created with a specific use case in mind in which clinicians work together with adult patients and want to visualize and analyze complex physiological data (Looff et al., 2021), as an additional tool for psychosocial information. Although clinicians indicated that the tool seems to provide an additional source of information in treatment, it needs to be further investigated whether the application has added value for their treatment, and what the added value is in other fields where wearables are used (Johnson and Picard, 2020). A second limitation is related to the usability of the Wearables package. Although the Shiny application has preset parameters from frequently used packages (Taylor et al., 2015; Martínez et al., 2017) and is thought to be easily utilized by non-experts, users should have some knowledge of programming in the R language (R Core Team, 2014). Knowledge of programming is necessary to change some of the settings from the Wearables package and might thus limit its usefulness. Related to this is the addition of new algorithms into the Shiny application. Interested users might download the package and Shiny application from Github and propose changes to the package. These could then be deployed in an updated version of the E4 dashboard and the Wearables package. However, if users want to incorporate these algorithms themselves, they should have considerable knowledge of functional and object-oriented programming, which might be a hurdle for users with limited programming experience. A solution to this problem might be to engage in a collective team science and citizen science approach in which patients, clinicians, science practitioners, and researchers work together to improve the usability and interpretability of the software.

Another limitation is that the Shiny application and R package are currently only available for the Empatica E4 (Garbarino et al., 2014). This limits the usability for other devices, although a modular and flexible architecture was chosen, so that the scripts could be easily extended to other wearables. In addition, although average HR can be calculated based on photoplethysmography (PPG) and also has predictive value in more complex heart rhythm analyses (Cheung et al., 2018), the PPG sensor is prone to artifacts and HRV measurements should, therefore, currently only be interpreted under resting conditions where motion is limited to an absolute minimum (van Lier et al., 2019; Schuurmans et al., 2020). In addition, it is unclear what the EDA signal specifically measures on the wrist as the validity of the EDA signal has recently been brought into question in comparison with more traditional finger measurements (Menghini et al., 2019; Milstein and Gordon, 2020). Further research is warranted to investigate the responsivity of EDA obtained from the wrist to stress and emotions and test whether wrist EDA has similar predictive and ecological validity as EDA obtained from the finger (Menghini et al., 2019). Recent research has also shown that the validity of the measures might also be dependent on the type of wearable, and the specific user that is targeted (Cosoli et al., 2020; Teixeira et al., 2021), so a careful investigation of these limitations is needed.

Related to these limitations are the reliability and validity of the algorithms to detect artifacts and extract features for HR and EDA. Both the EDA signal and HR signal have been used in previous validation studies comparing the physiological signals (and the derived features) to ground-truth devices (Menghini et al., 2019; van Lier et al., 2019; Milstein and Gordon, 2020; Schuurmans et al., 2020). These studies show that the signals can be used under specific conditions and for different levels of signal, parameter, and event features, but further validation is warranted. For instance, the study by Milstein and Gordon (2020) reported that the reliability of the EDA data is questionable in comparison with a ground truth device, while the study by van Lier et al. (2019) suggested that EDA might be useful for strong and sustained stressors. The HR (V) data seems to be useful under resting and movement conditions (Menghini et al., 2019; van Lier et al., 2019; Milstein and Gordon, 2020; Schuurmans et al., 2020). Deviations have also been reported between algorithms used for artifact detection and feature extraction (van Lier et al., 2019; Coffman et al., 2020; Schuurmans et al., 2020). A careful analysis of the reliability and validity of both the physiological signals and underlying algorithms for artifact detection and feature extraction is therefore direly needed.

### Directions for Future Research

Several directions for future research seem warranted. In the current Wearables package and Shiny application, only the artifact and peak detection methods for EDA by Taylor et al. (2015) are supported. The artifact detection method, developed by Taylor et al. (2015), uses a support vector machine classifier that was trained based on a physiological dataset that was labeled by experts. A recent publication (Coffman et al., 2020) claimed that the method by Taylor et al. (2015) might be considered too conservative in that it possibly discards too much data as an artifact. Future work could integrate the rule-based algorithm developed by Coffman et al. (2020) in the current Wearables package and Shiny application to compare both methods for EDA signal processing, both in the laboratory and real-life settings. Additionally, it would also be interesting to incorporate other methods to analyze EDA and HR data (Benedek and Kaernbach, 2010; Bach, 2014; Tarvainen et al., 2014; Chaspari et al., 2016).

As for the R language, both R and Python are open source languages and are frequently used by programmers, researchers, and data scientists to study a wide array of topics (Taylor et al., 2015; Depaoli et al., 2020; Qu et al., 2022). The current study integrated an R package with an R Shiny application (R Core Team, 2014; R Studio, 2021), but similar functionality is also available in Python. Future research could also focus on the integration of the current package into a Python library and web application (Nokeri, 2022).

Another important area of research could focus on integrating new algorithms for analyzing the blood volume pulse, on which the HR and IBI are based. Empatica currently does not disclose the second algorithm that is used to detect heartbeats from the BVP signal (Empatica support, 2021). Research can focus on the comparison of the results from both the Empatica method with newly developed methods to further develop the algorithms to detect heartbeats and calculate HR.

We mentioned utilizing a user-centered design approach for the development of the Shiny application based on studies that were conducted over the past years (de Looff P. et al., 2019; Looff et al., 2021). However, the current version of the Shiny application has had no formal validation on usability, acceptance, and continuous use intention. Therefore, future research is needed to assess the usability of the software.

Lastly, another interesting avenue for research is to compare predictive models that use raw physiological data as input and compare the results with processed and clean data. Researchers have the option to model the raw data from the signal directly (Goodwin et al., 2019) with different machine learning approaches (Cheung et al., 2018; Goodwin et al., 2019), as compared to research that analyses the cleaned and pre-processed data (de Looff P. C. et al., 2019; de Looff P. et al., 2019).

### Clinical Implications

Wearables seem to hold great potential for healthcare (Cheung et al., 2018; Johnson and Picard, 2020; Pedrelli et al., 2020) in disease monitoring (Regalia et al., 2019), including COVID-19 (Ates et al., 2021; Channa et al., 2021), predict risk for disease (Pedrelli et al., 2020), predict risk for dangerous behavior (de Looff P. et al., 2019; Goodwin et al., 2019; de Looff et al., 2022), or provide continuous feedback to stimulate emotional awareness and behavioral change (Derks et al., 2019). However, wearables also hold promise as an additional tool in the clinician’s current toolbox to provide opportunities for in-depth and careful investigation of physiological responses in daily life (Johnson and Picard, 2020). This might be especially useful if clinicians do not see their patients on a regular daily basis, and as a consequence, most of the patients’ physiological reactions in various daily life activities are obscured from the clinician. It needs to be carefully investigated if these applications could be used to increase valuable insights that might benefit the patients’ health and is effective for treatment.

The Shiny application and Wearables package are designed for clinicians and practitioners to discuss physiological reactivity in real-life and treatment settings with their patients. The Shiny application can also have value during therapy sessions to assess physiological reactions to a plethora of stimuli, as well as to assess whether patients show progressive physiological reactivity to trauma triggering emotions or to stimuli. Clinicians can now directly use the interface to select specific periods of interest and easily calculate all necessary physiological parameters to investigate the physiological reactions and changes over time (Crockett et al., 2017).

#### Clinical Use Cases

The benefit of discussing physiological reactivity in real-life offers additional tools to clinicians in understanding patients’ emotions and behavior in different real-life situations and might have extended value to the current clinical arsenal of questionnaires, assessments, and evaluations. To give an example from practice, following a weekend leave, patients are typically asked to evaluate how their weekend was and if they experienced unusual or stressful events. Clinicians, then, typically administer a standardized questionnaire to evaluate the psychological, social, emotional, or cognitive reactions that a patient might have had during the weekend. In one of our case studies, we observed that a patient showed heightened arousal evident in elevated levels of EDA and HR approximately 30 min preceding the patient’s return to the psychiatric hospital. It was assumed that the heightened arousal was due to the patient returning to the psychiatric hospital. After discussing the outcomes with the patient, it was later discovered that the patient was brought back to the psychiatric hospital by a family member, who had an alcohol-related problem, which resulted in the family member being drunk early in the morning. This made the patient feel very uncomfortable. Discussing the physiological reactions to a real-life situation provided additional information on the emotional state of the patient. In another example, a patient would typically watch the evening news in his chair. In discussing the physiological reactions with the patient, the practitioners noticed unusual patterns in physiological reactivity during the time that the patient was watching the news. The patient told the practitioner that the evening news was important to watch, but the patient was unsure what the events described in the news would mean for his current situation and the future. This provided insights into the emotional well-being of the patients and allowed the clinician to instigate an intervention. These examples illustrate use cases in which the physiological reactivity might provide a complementary source of information over and above the current arsenal of assessment options and, in which the downloadable report can be used as a talking board.

In sum, therefore, the current rise in the use of wearables (Cheung et al., 2018) offers new and insightful ways to bring neuroscientific research from the laboratory to practice and is expected to grow exponentially in the coming years (Johnson and Picard, 2020; de Looff et al., 2022). Careful consideration should be given to the accuracy, reliability, and validity of the (future) devices and physiological signals. The current Wearables package and Shiny application are first step in devising meaningful and valuable tools on an open-source platform to explore opportunities for a careful investigation of physiological reactions in daily life, while addressing common pitfalls in signal analysis (Taylor et al., 2015; Orphanidou, 2018; Menghini et al., 2019; van Lier et al., 2019; Coffman et al., 2020; Milstein and Gordon, 2020; Schuurmans et al., 2020).

## Data Availability Statement

The original contributions presented in the study are included in the article/supplementary material, further inquiries can be directed to the corresponding author.

## Ethics Statement

The study was granted approval by the scientific committees of the health organizations and committee of ethics of the Faculty of Social Sciences of Radboud University at Nijmegen (ECSW2019-005; 19U.501869) and conforms to the Declaration of Helsinki for ethical principles involving human participants. Written informed consent was obtained from all participants for their participation in this study.

## Author Contributions

PL: conceptualization, methodology, design, coding, writing manuscript, and validation. RD: methodology and coding. SK, KS, and MN: conceptualization, coding, review and editing, and validation. NJ and ST: coding, review and editing, and validation. FS: supervision and review and editing. All authors contributed to the article and approved the submitted version.

## Conflict of Interest

PL was employed by Fivoor B.V and De Borg. RD was employed by the company Shintō Labs. The remaining authors declare that the research was conducted in the absence of any commercial or financial relationships that could be construed as a potential conflict of interest.

## Publisher’s Note

All claims expressed in this article are solely those of the authors and do not necessarily represent those of their affiliated organizations, or those of the publisher, the editors and the reviewers. Any product that may be evaluated in this article, or claim that may be made by its manufacturer, is not guaranteed or endorsed by the publisher.

## Footnotes

1
https://github.com/PCdLf/e4dashboard

2
https://cran.r-project.org/web/packages/wearables/index.html

3
https://github.com/PCdLf/wearables
